# Partial Replacement of Dietary Fat with Polyunsaturated Fatty Acids Attenuates the Lipopolysaccharide-Induced Hepatic Inflammation in Sprague-Dawley Rats Fed a High-Fat Diet

**DOI:** 10.3390/ijerph182010986

**Published:** 2021-10-19

**Authors:** Hee-Kyoung Son, Huo Xiang, Seohyun Park, Jisu Lee, Jae-Joon Lee, Sunyoon Jung, Jung-Heun Ha

**Affiliations:** 1 Research Center for Industrialization of Natural Neutralization, Dankook University, Cheonan 31116, Korea; kyoung1033@dankook.ac.kr (H.-K.S.); waitfor0126@naver.com (H.X.); sb5590@naver.com (S.P.); dlwltn970811@naver.com (J.L.); 2 Department of Food Science and Nutrition, Dankook University, Cheonan 31116, Korea; 3 Department of Food and Nutrition, Chosun University, Gwangju 61452, Korea; leejj80@chosun.ac.kr

**Keywords:** high-fat diet, lipopolysaccharide, polyunsaturated fatty acids, perilla oil, corn oil, inflammation

## Abstract

In this study, we investigated whether the partial replacement of dietary fat with polyunsaturated fatty acids (PUFAs) ameliorated the lipopolysaccharide (LPS)-induced hepatic inflammation in rats fed a high-fat diet. Male Sprague-Dawley rats were divided into three groups and provided each of the following diets: (1) high-fat diet (HFD), (2) HFD with perilla oil (PO), and (3) HFD with corn oil (CO). After 12 weeks of dietary intervention, the rats were intraperitoneally injected with LPS (5 mg/kg) from *Escherichia coli* O55:B5 or phosphate-buffered saline (PBS). Following LPS stimulation, serum insulin levels were increased, while PO and CO lowered the serum levels of glucose and insulin. In the liver, LPS increased the triglyceride levels, while PO and CO alleviated the LPS-induced hepatic triglyceride accumulation. In the LPS injected rats, the mRNA expression of genes related to inflammation and endoplasmic reticulum (ER) stress was attenuated by PO and CO in the liver. Furthermore, hepatic levels of proteins involved in the nuclear factor kappa-light-chain-enhancer of activated B cells/mitogen-activated protein kinase pathways, antioxidant response, and ER stress were lowered by PO- and CO-replacement. Therefore, the partial replacement of dietary fat with PUFAs alleviates LPS-induced hepatic inflammation during HFD consumption, which may decrease metabolic abnormalities.

## 1. Introduction

Obesity is defined as the accumulation of excess body fat, which may have adverse effects on health [1]. Obesity increases the risk of various metabolic complications, including type 2 diabetes, heart disease, and cardiovascular diseases [2,3]. In addition, nonalcoholic fatty liver disease (NAFLD) is becoming the most common cause of chronic liver disease due to the increasing prevalence of obesity [4]. Steatosis, a hallmark of NAFLD, occurs when hepatic fatty acid uptake increases due to high energy intake [4]. Previous studies have shown that it contributes to systemic insulin resistance and inflammatory responses, which may occur in obesity [5]. The probability of developing hepatic fibrosis and hepatocellular carcinoma is greater in individuals with steatohepatitis. Lipopolysaccharide (LPS) is considered a potent inducer of hepatic inflammation in experimental rodents [6,7]. LPS is reported to be the predominant cause of hepatic neutrophil infiltration in steatohepatitis patients [6]. To date, a few studies have examined how obese mouse models respond to acute inflammatory stimuli. They have revealed that, in response to LPS stimulation, obese rodents showed increased cytokine expression and more severe liver dysfunction and insulin resistance compared to lean subjects [7,8].

The onset of obesity is multifactorial, including genetic, environmental, dietary, and environmental factors [2,9]; however, the most common cause of obesity is that energy intake is greater than energy consumption. Nowadays, people tend to consume high-fat and/or high-sugar diets and have less energy expenditure, such as lower physical activity [9,10]. Furthermore, recently, the spread of COVID-19 throughout the world has triggered many physical restrictions when compared with any other period in human history, and thus individuals worldwide are at a higher risk of being overweight and developing obesity [11]. Therefore, appropriate dietary selection, including fatty acids, is highly recommended. The World Health Organization (WHO), China, and the United States have recommended reducing fat intake to less than 30% of the total energy intake to prevent obesity [12]. Moreover, the WHO recommends reducing the saturated fat intake to less than 10% and the trans-fat intake to less than 1% of the total energy intake, respectively, to reduce the onset risk of cardiovascular diseases [13]. Therefore, consuming alternative and functional fat sources may be an important dietary strategy, such as replacing saturated and/or trans-fat intake with polyunsaturated fatty acids (PUFAs) to prevent metabolic complications [13,14].

PUFAs are fatty acids that contain more than one double bond in their backbone. PUFAs are categorized into omega-3 (*n*-3) and omega-3 (*n*-6) PUFAs according to their chemical structure [15]. The *n*-3 desaturase is not sufficient in mammalian cells, and therefore, it cannot efficiently convert *n*-6 fatty acids to *n*-3 fatty acids; therefore, certain *n*-3 fatty acids are essential for mammals. The three types of *n*-3 fatty acids engaged in human physiology are α-linolenic acid (ALA), eicosapentaenoic acid (EPA), and docosahexaenoic acid (DHA). ALA is commonly found in plant oils, and EPA and DHA are mainly present in fish and shellfish [15]. Although the recommended daily intake of *n*-3 fatty acids varies across international organizations, consuming >250 mg *n*-3 fatty acids is highly preferable [16,17]. The ratio of *n*-6 to *n*-3 fatty acids is another crucial factor. Previous studies have revealed that an *n*-6/*n*-3 PUFA diet with a ratio of 6−10 may possess various health benefits [18]. Unfortunately, the Western diet generally has lower *n*-3 fatty acids, and the ratio of *n*-6/*n*-3 is 15/1–16.7/1. This excessive intake of *n*-6 PUFAs may trigger the pathogenesis of inflammation, autoimmune diseases, cardiovascular disease, and cancer [19,20]. Therefore, balanced dietary fatty acid supplementation is highly important to date [21].

In a previous study, a diet rich in saturated fat was associated with higher production of pro-inflammatory cytokines, particularly in overweight or diabetic individuals [22,23]. However, the anti-inflammatory capacity of PUFA-rich diets has been well documented [24,25]. Therefore, this study was conducted to investigate the preventive effect of PUFA on LPS-induced hepatic inflammation in high-fat diet-induced obese Sprague-Dawley rats. In the present study, we supplemented perilla oil (PO) or corn oil (CO) in a high-fat diet to partly replace saturated fat with PUFAs. PO is a rich source of *n*-3 PUFAs, such as ALA, EPA, and DHA [26]. In previous studies, PO was reported to reduce insulin resistance [27] and regulate lipid metabolism [28], intestinal flora [29], and colitis [30]. On the other hand, corn oil (CO) is a widely consumed edible oil that contains up to 80% unsaturated fatty acids and is an abundant source of linoleic acid (LA, *n*-6).

## 2. Materials and Methods

### 2.1. Animal Experiments and Diets

All animal studies were approved by the Dankook University Institutional Animal Care and Use Committee (IACUC, No. DKU-19-031). In total, 48 male (5-week-old) Sprague-Dawley rats were obtained from DooYeal Biotech (Seoul, Korea). The rats were housed in shoe-box cages in a temperature- and humidity-controlled (23 ± 2 °C, 55 ± 5%) room with a 12 h light/dark cycle. The experimental animals had free access to the diet and purified water. After acclimatization for one week, the rats were randomly assigned to three different diets: (1) high-fat diet (HFD), (2) HFD + perilla oil (HFD + PO), and (3) HFD + corn oil (HFD + CO). PO (CJ Cheil Jedang Co., Seoul, Korea) and CO (CJ Cheil Jedang Co.) were purchased from a local market and stored at −20 °C. The amounts of PO and CO added were determined to meet human requirements for *n*-3 and *n*-6 PUFAs by converting animal intake into human intake [31,32]. The experimental diets were formulated based on the AIN-93G diet with slight modification [33]. The constant and variable compositions of each experimental diet are presented in Table 1 and Table 2, respectively. After 12 weeks of dietary intervention, the experimental rats were intraperitoneally injected with either phosphate-buffered saline (PBS) or 5 mg/kg of LPS (*Escherichia coli*, O55:B5; Sigma-Aldrich, St. Louis, MO, USA) under overnight fasting conditions. Next, the experimental rats were euthanized by carbon dioxide (CO_2_) exposure followed by cervical dislocation. Whole blood was collected by cardiac puncture, maintained at room temperature for 30 min, and centrifuged (3000× *g*, 15 min) to obtain serum. Liver and multiple white adipose tissue (WAT) fat pads were harvested, weighed, and snap-frozen quickly in liquid nitrogen. Serum and tissue samples were stored in a deep freezer set at −70 °C for further analysis.

### 2.2. Fatty Acid Composition Analysis of Experimental Diets

The fatty acid composition of the experimental diets was assessed by methyl esterification of boron trifluoride (BF_3_)-methanol [34]. First, dietary samples (~0.1 g) were added to a test tube, followed by hexadecanoic acid (C17:0) (0.5 mL, 1 mg/mL hexane). Then, 0.5 N NaOH-methanol (2 mL) was added, and the mixture was heated for 10 min at 110 °C with a heating/stirring module (Thermo Fisher Scientific, Waltham, MA, USA) using Reacti-Therm III. The mixture was then cooled to room temperature, with the subsequent addition of BF_3_-methanol (4 mL). The mixture was heated at 110 °C for 1 h and then cooled. Next, hexane (2 mL) was added, and the mixture was stirred for 1 min, and the hexane layer was collected for lipid analysis. For detection, a gas chromatography (GC) flame ionization detector (FID) with an Agilent Technologies 6890 N instrument was employed, and helium was used as the carrier gas (2.7 mL/min) with an SP-2560 capillary column (Agilent Technologies, Santa Clara, CA, USA). The temperature of the sample injector and detector was set to 250 °C, and the splitting ratio was set to 50:1. The flow rates of air and hydrogen ionized by the flame were 450 and 40 mL/min, respectively. The initial temperature of the oven was set to 130 °C for 5 min. The temperature was then increased by 4 °C/min up to 240 °C and continued for 15 min. All analyses were conducted in triplicate. The results were evaluated by comparing with the standard fatty acid reference (Supelco 37 FAME; Sigma-Aldrich, Sigma-Aldrich, St. Louis, MO, USA).

### 2.3. Oral Glucose Tolerance Test (OGTT)

OGTT was conducted at weeks 3, 7, and 11. The day before the OGTT, the rats were fasted overnight. During the fasting period, only drinking water was provided. Animals were orally administered glucose (1 g/kg body weight) by tube feeding, and blood glucose levels were measured by drawing blood from the tail vein and then using a blood glucose meter (Accu-Chek; Roche Diagnostics, Basel, Switzerland). Subsequently, the rat blood glucose levels were measured at 0, 15, 30, 60, and 120 min.

### 2.4. Fatty Acid Composition Analysis of Whole Blood

The fatty acid composition of whole blood was measured in three pooled samples due to the sample volume limitations. Samples collected from two to three animals in each group were included in any one pool. To ensure that no individual sample greatly influenced the pooled results, the sample volume of whole blood collected from each animal was of equal. For the analysis of the fatty acid composition, a drop of whole blood samples was spiked on a blood-spot card pre-coated with an antioxidant mixture. The card was purchased from OmegaQuant (Sioux Falls, SD, USA). The blood fatty acid composition was analyzed by gas chromatography (GC) as described by Jackson et al. [35]. The fatty acid composition was expressed as a percentage of the total identified fatty acids.

### 2.5. Biochemical Analyses of Serum

The serum levels of triglyceride (TG), total cholesterol (TC), high-density lipoprotein cholesterol (HDL-C), aspartate aminotransferase (AST), alanine aminotransferase (ALT), and alkaline phosphatase (ALP) were measured using commercial kits (Embiel, Gunpo, Korea). To obtain the non-HDL-C level, we subtracted HDL-C levels from the TC level. The atherogenic coefficient (AC) was calculated using the following formula: AC = TC − HDL-C/HDL-C and the cardiac risk factor (CRF) was calculated using the following formula: CRF = TC/HDL-C [36]. Serum glucose levels were analyzed using a rat glucose test kit (Crystal Chem, Downers Grove, IL, USA), and serum insulin levels were analyzed using an enzyme-linked immunosorbent assay (ELISA) reagent kit (Mercodia, Uppsala, Sweden). The homeostasis model assessment-estimated insulin resistance (HOMA-IR) value was calculated as follows: HOMA-IR = fasting insulin in μU/L × fasting glucose in mg/dL/405 [37]. Serum leptin levels were determined using the MILIPLEX map Luminex assay (Millipore, Burlington, MA, USA).

### 2.6. Determination of Lipid Contents in the Liver and Adipose Tissue

Hepatic and epididymal adipose tissue (EAT) lipids were extracted using the method described by Bligh and Dyer methods [38], with slight modifications. The concentrations of TG and TC were determined using commercial kits (Embiel), and the results were expressed as mg per g of tissue weight.

### 2.7. Quantitative Reverse Transcription-Polymerase Chain Reaction (qRT-PCR)

Total RNA was isolated from liver tissue using NucleoZoL Reagent (Macherey-Nagel, GmbH & Co. KG, Düren, Germany) and DNA-free™ Kit (Thermo Fisher Scientific). The isolated RNA was quantitatively analyzed using a SpectraDrop™ Micro-Volume Microplate (SpectraMax iD3; Molecular Devices, San Jose, CA, USA). Next, cDNA was synthesized from 1 μg of total RNA using the iScript™ cDNA Synthesis Kit (Bio-Rad Laboratories, Hercules, CA, USA), with the T100 Thermal Cycler (Bio-Rad Laboratories) set to priming (25 °C, 5 min), reverse transcription (46 °C, 20 min), RT inactivation (95 °C, 1 min), and then diluted twice with the Ambion™ Nuclease-Free Water (Thermo Fisher Scientific). qRT-PCR was performed using the CFX Connect Real-Time PCR Detection System (Bio-Rad Laboratories). The primer sequences used in this study are listed in Table 3. The cDNA sample (2 µL), 10 µM forward primer (Macrogen, Seoul, Korea) (1 µL), 10 µM reverse primer (Macrogen) (1 µL), Ambion™ Nuclease-Free Water (5 µL), 2× PCR Master Mix (10 µL), 20× SFCGreen^®^ I Dye (BioFACT, Daejeon, Korea) (1 µL), and mixture at a final total volume of 20 µL were injected into each well of Multiplate™ 96-Well PCR plates (Bio-Rad Laboratories). Then, a Microseal ‘B’ PCR plate sealing film (Bio-Rad Laboratories) was affixed before the reaction. DNA denaturation (95 °C, 3 min) was performed for polymerase activation by thermal cycling. Subsequently, denaturation (95 °C, 10 s), annealing/extension (55 °C, 30 s), and the plate read were set, and go to 2 was performed in a circular fashion (49 times). After completion of the reaction, quantitative analysis was performed using CFX Maestro (Bio-Rad Laboratories). Glyceraldehyde 3-phosphate dehydrogenase (*GAPDH*), a housekeeping gene, was used as the internal control [39]. The expression level of *GAPDH* did not significantly vary between the groups. The relative mRNA level (fold change) was calculated and compared with that of the HFD control group.

### 2.8. Western Blot Analysis

Liver tissue was homogenized using an ultrasonic cell disruptor (Branson Sonifier^®^; Branson, Danbury, CT, USA) with a mixture of ice-cold radioimmunoprecipitation assay (RIPA) lysis buffer (ATTO, Japan) 99: Halt™ Protease and Phosphatase Inhibitor Single-Use Cocktail 100× (Thermo Fisher Scientific) 1, followed by slow stirring at 4 °C (2 h) and centrifugation at 16,000× *g* at 4 °C for 20 min. Total protein lysates were collected from the supernatants. The protein content of the extract was determined using a Pierce™ BCA Protein Assay Kit (Thermo Fisher Scientific). Based on the obtained results, a 30 µg/µL protein mixture was prepared by mixing sodium dodecyl sulfate (SDS) loading buffer 5× (6 µL) and distilled water with the extract. The proteins were separated by 10–12% SDS-polyacrylamide gel electrophoresis (SDS-PAGE) and then transferred onto a polyvinylidene difluoride (PVDF) membrane (Bio-Rad Laboratories). The membrane was blocked with 5% skim milk (Becton-Dickinson, Franklin Lakes, NJ, USA) and incubated at 4 °C overnight with primary antibodies, followed by incubation with the corresponding secondary antibodies. The antibodies used for Western blotting are presented in Table 4. Finally, the samples were stained with SuperSignal™ West Pico PLUS Chemiluminescent Substrate (Thermo Fisher Scientific), scanned using Chemidoc (Davinch-Western™ Imaging system; Davinch-K, Seoul, Korea) for graphical results, and then quantitatively analyzed using the Image J Software v.1.8 (National Institutes of Health, Bethesda, MD, USA). β-Actin was used as an internal control. Finally, the protein expression level (fold change) was calculated by comparing the HFD and control groups.

### 2.9. Statistical Analysis

Values are expressed as the mean ± standard deviation (SD) or Box-and-Whisker-plots. Graphs were prepared using GraphPad Prism 5 for Windows (GraphPad Software Inc., San Diego, CA, USA). Data were analyzed using one- or two-way analysis of variance (ANOVA), followed by Tukey’s post hoc test using XLSTAT 2012 for windows (Addinsoft Inc., Paris, France). Statistical significance was set at *p* < 0.05. A summary of the statistical results of the two-way ANOVA for main effects and interactions is presented in Table 5.

## 3. Results

### 3.1. Fatty Acid Profiles of Experimental Diets and Rat Whole Blood

The fatty acid composition of the experimental diets is presented in Table 6. The saturated fatty acid (SFA) content was high in the order of HFD, HFD + PO, and HFD + CO groups, but the MUFA content was lower in the HFD group than in the HFD + PO and HFD + CO groups. On the other hand, the PUFA content was high in the order of HFD + CO, HFD + PO, and HFD groups. Among them, the *n*-6 fatty acid content was the highest in the HFD + CO group, whereas the *n*-3 fatty acid content was the highest in the HFD + PO group. Due to the high content of *n*-3 fatty acids, the *n*-6/*n*-3 ratio of HFD + PO (0.95) was considerably lower than that of HFD + CO (62.24).

Table 7 shows the fatty acid composition of the whole blood of rats after dietary intervention. In rats of the HFD + PO group, the concentrations of *n*-3 fatty acids, such as α-linolenic acid (ALA), eicosapentaenoic acid (EPA), docosapentaenoic acid (DPA), and docosahexaenoic acid (DHA), were markedly higher than those in the rats in the HFD + CO group. In contrast, in the HFD + CO group, the concentrations of *n*-6 fatty acids, such as linoleic acid (LA), arachidonic acid (AA), docosatetraenoic acid (DTA), and docosapentaenoic acid (DPA), were significantly higher than those in the HFD + PO group. Consequently, the *n*-6/*n*-3 ratio of the whole blood in the HFD + PO group was markedly ~5 times lower than that in the HFD + CO group.

### 3.2. Partial Replacement of Dietary Fat with PO and CO Did Not Affect the Body Weight and Food Intake

**Figure 1:**
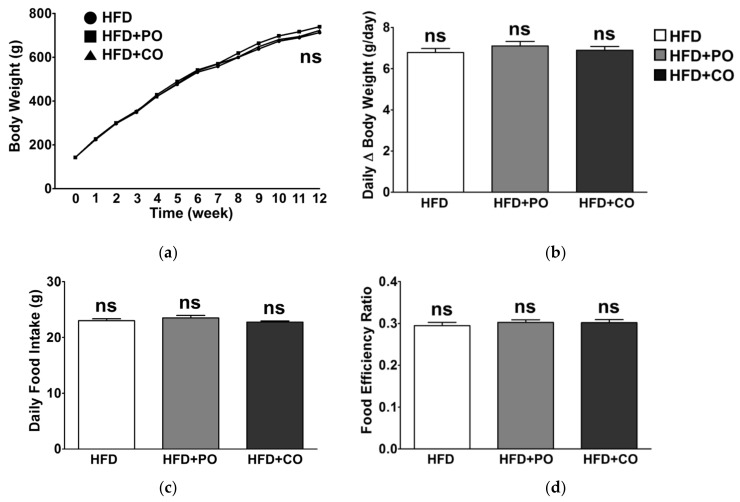
Effects of partial replacement of dietary fat with PO or CO on the body weight and food intake of rats. Five-week-old male Sprague-Dawley rats were fed either a high-fat diet (HFD), HFD supplemented with perilla oil (HFD + PO), or corn oil (HFD + CO) for 12 weeks (*n* = 16 per group). (**a**) Body weight changes; (**b**) daily body weight gain; (**c**) daily food intake; (**d**) food efficiency ratio (FER). Values are presented as the mean ± standard deviation. Data were analyzed using one-way analysis of variance (ANOVA) and Tukey’s multiple comparisons post hoc test for multiple comparisons. ‘ns’ indicates *p* ≥ 0.05.

In this study, rats were fed an experimental diet *ad libitum*. During the 12 weeks of experimental periods, the body weight of all groups gradually increased due to high-fat feeding; however, no noticeable changes in body weight and daily body weight gain were observed among the groups (Figure 1a,b). Daily food intake, calculated by dividing the total dietary intake by the experimental period, did not significantly differ among the groups (Figure 1c). Thus, there were no significant changes in the food efficiency ratio among the groups (Figure 1d). Therefore, partial replacement of high fat with PUFAs did not affect body weight, food intake, and food efficiency in our animal experimental settings.

### 3.3. Partial Replacement of Dietary Fat with PO and CO Improved Glucose Metabolism

**Figure 2:**
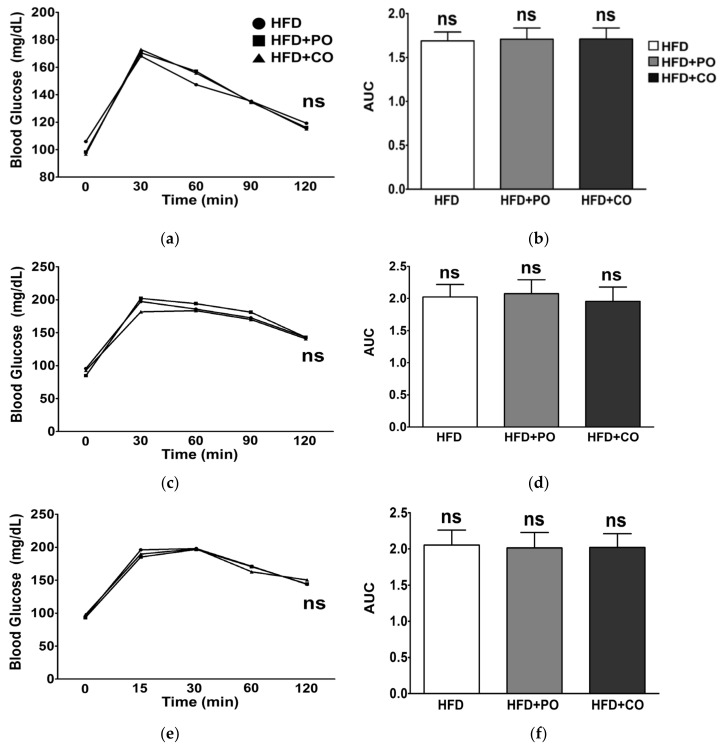
Effects of partial replacement of dietary fat with PO or CO on oral glucose tolerance test (OGTT). Five-week-old male Sprague-Dawley rats were fed either the HFD, HFD + PO, or HFD + CO for 12 weeks (*n* = 16 per group). (**a**,**c**,**e**) OGTT at weeks 3, 7, and 11; (**b**,**d**,**f**) area under the curve (AUC) at weeks 3, 7, and 11 following OGTT. Values are represented by the line or as the mean ± standard deviation. Data were analyzed using one-way ANOVA and Tukey’s multiple comparisons post hoc test for multiple comparisons. ‘ns’ indicates *p* ≥ 0.05.

During the experimental period, we conducted an OGTT to evaluate whether the partial replacement of dietary fat with PO and CO improves glucose utilization. In previous studies, substituting SFA with PUFA was shown to improve glucose sensitivity in rodents and humans [40,41]. At weeks 3, 5, and 11, blood glucose levels peaked at 30 min after glucose administration; however, the recovery rate of blood glucose level at weeks 5 and 11 seemed to be slower than at week 3 (Figure 2a,c,e). There were no significant differences in glucose levels among the groups at any other time point (Figure 2a,c,e). In addition, the area under the curve (AUC) at weeks 3, 7, and 11 were not significantly different following OGTT (Figure 2b,d,f). 

**Figure 3:**
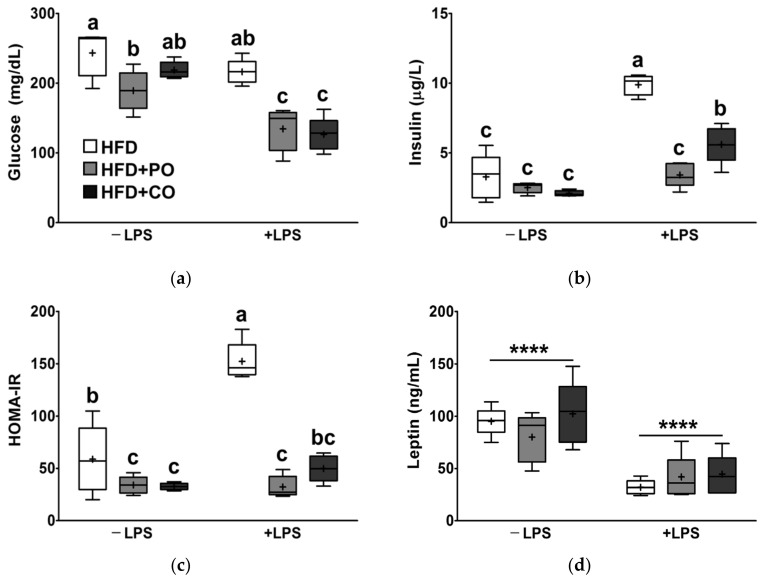
Effects of partial replacement of dietary fat with PO or CO and LPS stimulation on serum glucose, insulin, and leptin levels. Five-week-old male Sprague-Dawley rats were fed either the HFD, HFD + PO, or HFD + CO for 12 weeks and then treated with PBS or LPS (5 mg/kg) for 24 h (*n* = 8 per group). (**a**) Glucose level; (**b**) insulin level; (**c**) homeostasis model assessment-estimated insulin resistance (HOMA-IR); (**d**) leptin level. Data were analyzed using two-way ANOVA and Tukey’s multiple comparisons post hoc test for multiple comparisons. Means with different letters indicate significant differences at *p* < 0.05. Asterisk indicates a significant main effect for LPS (**** *p* < 0.0001). LPS, lipopolysaccharide; PBS, phosphate-buffered saline.

Fasting glucose and insulin levels are critical predictors of diabetes. Leptin is an adipokine that plays a pivotal role in regulating glucose metabolism. Following LPS treatment, fasting glucose levels tended to be reduced (Figure 3a), while the fasting insulin level and insulin resistance index (HOMA-IR) were significantly elevated (Figure 3b,c). Interestingly, in rats injected with LPS, fasting glucose and insulin levels were significantly lower in the HFD + PO and HFD + CO groups than in the HFD group (Figure 3a,b). In particular, HOMA-IR was considerably lower in the HFD + PO and HFD + CO groups than in the HFD group, regardless of LPS stimulation (Figure 3c). Furthermore, following LPS treatment, serum leptin levels were markedly reduced, whereas serum leptin levels were not profoundly affected by the diet (Figure 3d). 

### 3.4. Partial Replacement of Dietary Fat with PO and CO Amplified the Changes in Serum Lipids Induced by LPS

**Figure 4:**
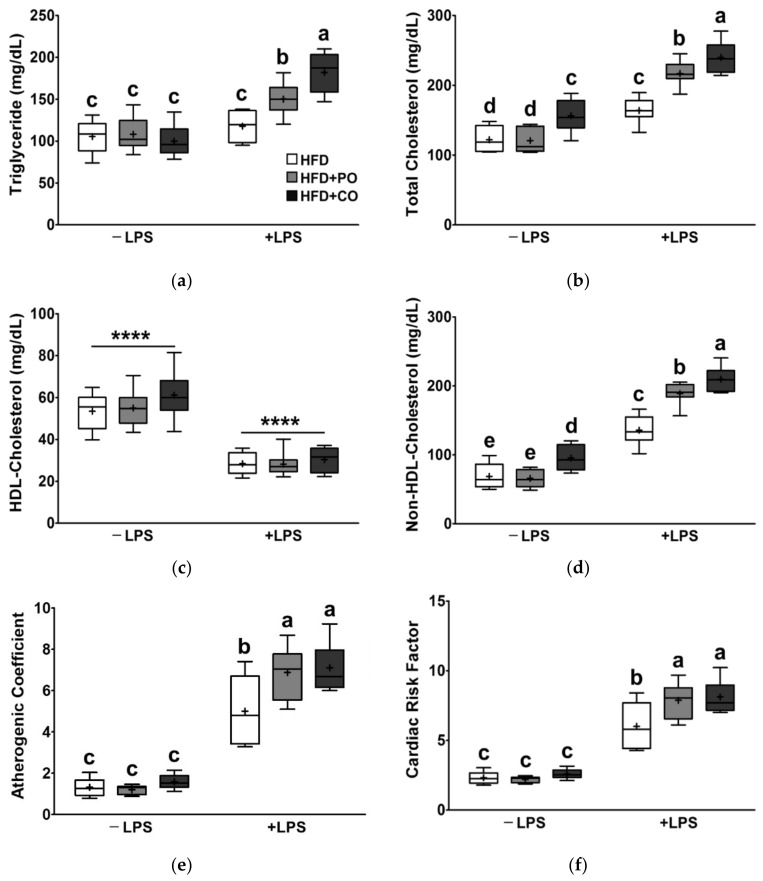
Effects of partial replacement of dietary fat with PO or CO and LPS stimulation on the serum lipid profile. Five-week-old male Sprague-Dawley rats were fed either the HFD, HFD + PO, or HFD + CO for 12 weeks and then treated with PBS or LPS (5 mg/kg) for 24 h (*n* = 8 per group). (**a**) Serum triglyceride (TG) level; (**b**) serum total cholesterol (TC) level; (**c**) high-density lipoprotein (HDL)-cholesterol level; (**d**) non-HDL-cholesterol level; (**e**) atherogenic coefficient; (**f**) cardiac risk factor. Data were analyzed using two-way ANOVA and Tukey’s multiple comparisons post hoc test for multiple comparisons. Means with different letters indicate significant differences at *p* < 0.05. Asterisk indicates a significant main effect for LPS (**** *p* < 0.0001). LPS, lipopolysaccharide; PBS, phosphate-buffered saline.

As the blood lipid profile changes in response to dietary fat, we determined the effects of HFDs supplemented with PO or CO, combined with LPS stimulation, on serum lipid levels. Following LPS treatment, the serum levels of TC, non-HDL-C, AC, and CRF were remarkably increased (Figure 4b,d–f). On the other hand, HDL-C levels decreased in the presence of LPS (Figure 4c). Prior to LPS treatment, serum levels of TC and non-HDL-C were significantly higher in the HFD + CO group than in the HFD group (Figure 4b,d). Compared with the HFD group, the levels of serum TG, TC, non-HDL-C, AC, and CRF were increased in the HFD + PO and HFD + CO groups following LPS treatment (Figure 4a,b,d–f).

### 3.5. Partial Replacement of Dietary Fat with PO and CO Altered the Liver Function Parameter Changes Induced by LPS

**Figure 5:**
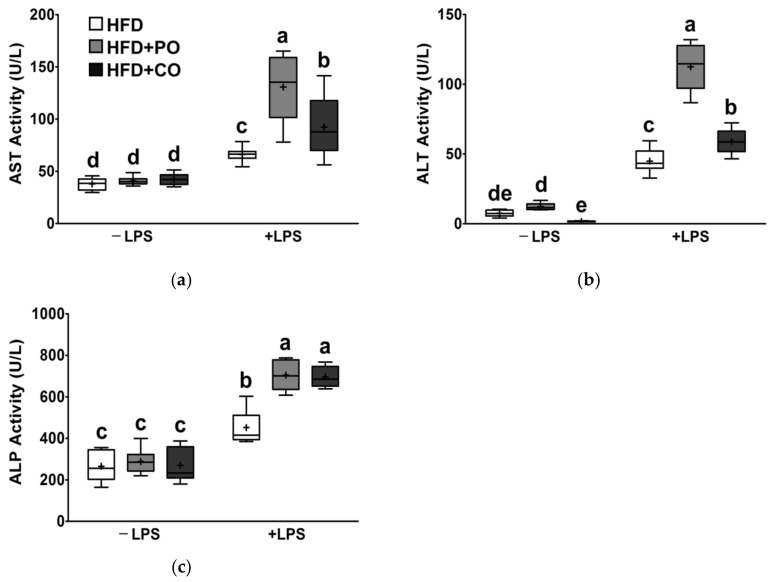
Effects of partial replacement of dietary fat with PO or CO and LPS stimulation on liver function parameters in serum. Five-week-old male Sprague-Dawley rats were fed either the HFD, HFD + PO, or HFD + CO for 12 weeks and then treated with PBS or LPS (5 mg/kg) for 24 h (*n* = 8 per group). (**a**) Aspartate aminotransferase (AST) activity; (**b**) alanine aminotransferase (ALT) activity; (**c**) alkaline phosphatase (ALP) activity. Data were analyzed using two-way ANOVA and Tukey’s multiple comparisons post hoc test for multiple comparisons. Means with different letters indicate significant differences at *p* < 0.05. LPS, lipopolysaccharide; PBS, phosphate-buffered saline.

Next, we determined the effects of HFDs supplemented with PO or CO, combined with LPS stimulation, on hepatic function parameters (AST, ALT, and ALP) in rats. After LPS treatment, serum levels of AST, ALT, and ALP were significantly increased by 2.39-fold, 9.97-fold, and 2.24-fold, respectively, compared with the HFD group, AST, ALT, and ALP levels were higher in the HFD + PO and HFD + CO groups (Figure 5a–c). Without LPS treatment, serum AST, ALT, and ALP levels were not changed by the HFD + PO and HFD + CO diets (Figure 5a–c).

### 3.6. Partial Replacement of Dietary Fat with PO and CO Did Not Alter the Liver and White Adipose Tissue Weight (WAT)

**Figure 6:**
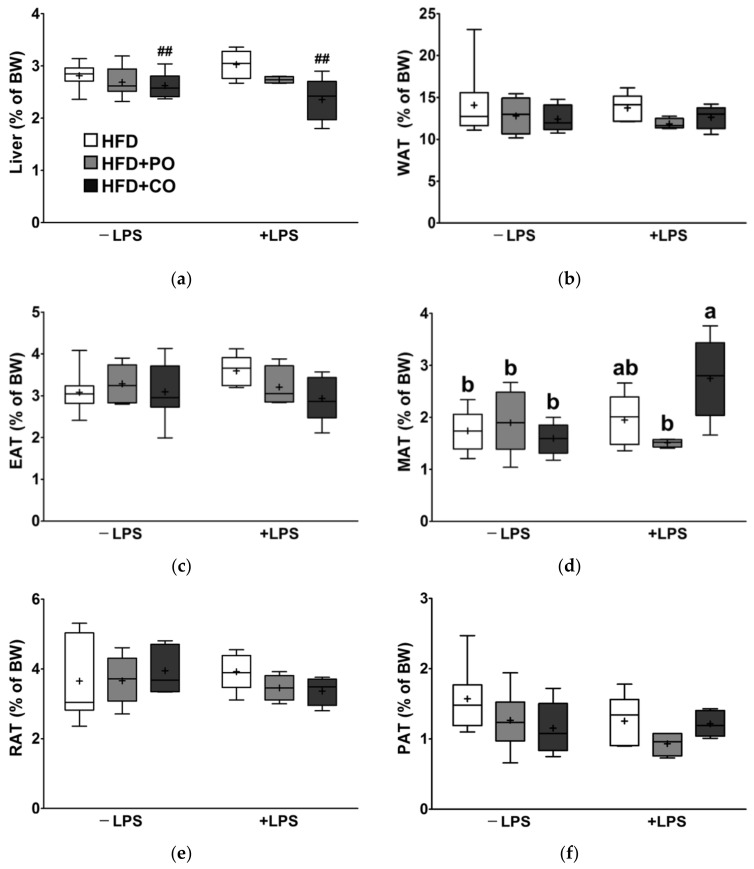
Effects of partial replacement of dietary fat with PO or CO and LPS stimulation on the relative weights of liver and white adipose tissue (WAT). Five-week-old male Sprague-Dawley rats were fed either the HFD, HFD + PO, or HFD + CO for 12 weeks, followed by treatment with PBS or LPS (5 mg/kg) for 24 h (*n* = 8 per group). (**a**) Liver weight; (**b**) WAT weight; (**c**) epididymal adipose tissue (EAT) weight; (**d**) mesenteric adipose tissue (MAT) weight; (**e**) retroperitoneal adipose tissue (RAT) weight; (**f**) perirenal adipose tissue (PAT) weight. Data were analyzed using two-way ANOVA and Tukey’s multiple comparisons post hoc test for multiple comparisons. Means with different letters indicate significant differences at *p* < 0.05. Hash indicates a significant main effect for diet (^##^ *p* < 0.01). LPS, lipopolysaccharide; PBS, phosphate-buffered saline; BW, body weight.

The liver and adipose tissues are the central metabolic organs. Therefore, the weights of the liver, total WAT (the sum of the weight of EAT, mesenteric adipose tissue (MAT), retroperitoneal adipose tissue (RAT), and perirenal adipose tissue (PAT)), and individual WAT were measured and expressed as a percentage of the body weight. There were no marked changes in the liver weight and WAT weight in the present study after LPS stimulation (Figure 6a–f). Partial replacement of dietary fat with CO reduced liver weight regardless of LPS challenge (Figure 6a). However, the WAT weights did not significantly change with PO and CO supplementation (Figure 6b–f). 

### 3.7. Partial Replacement of Dietary Fat with PO and CO Mitigated the LPS-Induced Lipid Changes in Liver and EAT

**Figure 7:**
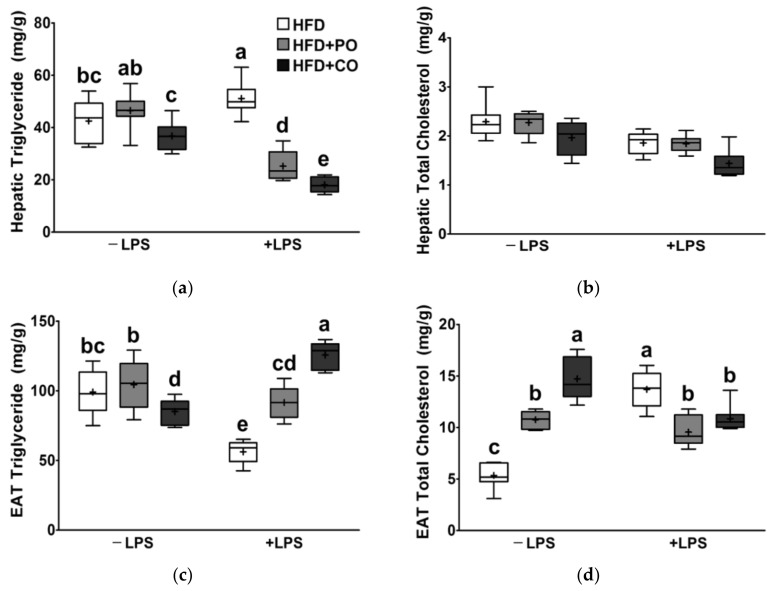
Effects of partial replacement of dietary fat with perilla oil or corn oil and LPS stimulation on lipid contents in liver and epididymal adipose tissue (EAT). Five-week-old male Sprague-Dawley rats were fed either the HFD, HFD + PO, or HFD + CO for 12 weeks and then treated with PBS or LPS (5 mg/kg) for 24 h (*n* = 8 per group). (**a**) Hepatic TG level; (**b**) hepatic TC level; (**c**) TG level in EAT; (**d**) TC level in EAT. Data were analyzed using two-way ANOVA and Tukey’s multiple comparisons post hoc test for multiple comparisons. Means with different letters indicate significant differences at *p* < 0.05. LPS, lipopolysaccharide; PBS, phosphate-buffered saline.

Next, we assessed the effects of dietary high fat replacement and LPS stimulation on TG and TC levels in the liver and EAT. Prior to LPS treatment, hepatic TG levels of the HFD + PO and HFD + CO groups did not differ from those of the HFD group; however, the HFD + CO group had significantly lower hepatic TG levels than the HFD + PO group (Figure 7a). In the HFD group, hepatic TG levels were significantly increased following LPS treatment, whereas the HFD + PO and HFD + CO groups showed significantly lower hepatic TG levels compared with the HFD group, and the level of HFD + CO was much lower than that of the HFD + PO group (Figure 7a). Hepatic TC levels were not changed by either dietary or LPS treatment (Figure 7b).

Before LPS treatment, the TG level of EAT in the HFD + PO group was not different from that in the HFD group; however, the HFD + CO group had a significantly lower TG level than the HFD + PO group (Figure 7c). Following LPS stimulation, EAT TG levels were decreased in the HFD group; however, compared with the HFD group, the HFD + PO and HFD + CO groups had higher TG levels, which was even higher in the HFD + CO group (Figure 7c). EAT TC levels in the HFD + PO and HFD + CO groups before LPS treatment were significantly higher than those in the HFD group (Figure 7d). In the HFD group, TC levels in the EAT were elevated considerably after LPS treatment, but the EAT TC levels in the HFD + PO and HFD + CO groups were significantly lower than those in the HFD group (Figure 7d).

### 3.8. Partial Replacement of Dietary Fat with PO and CO Alleviated the mRNA Expression of Genes Related to Inflammation and ER Stress in the Liver of Rats Injected with LPS

**Figure 8:**
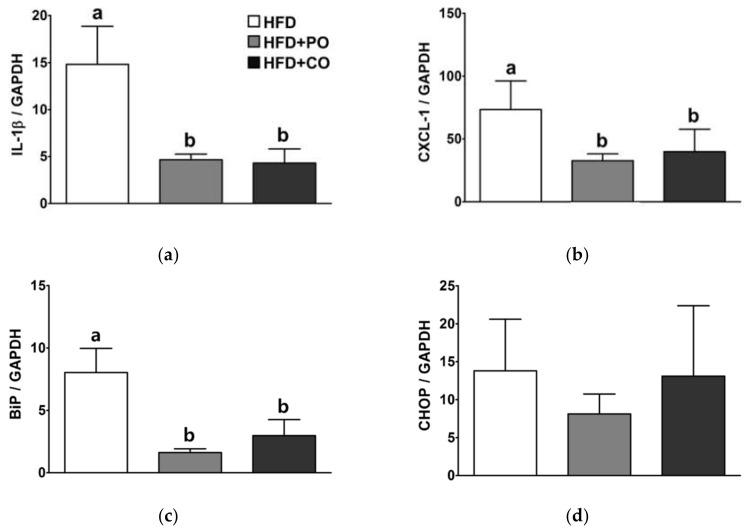
Effects of partial replacement of dietary fat with PO or CO and LPS stimulation on the mRNA expression of genes related to inflammation and endoplasmic reticulum (ER) stress in the liver. Five-week-old male Sprague-Dawley rats were fed either the HFD, HFD + PO, or HFD + CO for 12 weeks and were then treated with LPS (5 mg/kg) for 24 h (*n* = 8 per group). (**a**) Interleukin (IL)-1β level; (**b**) chemokine (C-X-C motif) ligand 1 (CXCL1) level; (**c**) binding immunoglobulin protein (BiP) level; (**d**) C/EBP homologous protein (CHOP) level. Relative expression of each gene was quantified by using the 2^−ΔΔCT^ method. Glyceraldehyde 3-phosphate dehydrogenase (*GAPDH*) was used as the reference gene for normalization. Values are presented as the mean ± standard deviation. Data were analyzed using one-way ANOVA and Tukey’s multiple comparisons post hoc test for multiple comparisons. Means with different letters indicate significant differences at *p* < 0.05. LPS, lipopolysaccharide.

Fatty liver and ER stress are reciprocally associated with the induction of hepatic inflammation. Therefore, we investigated the effects of HFDs supplemented with PO or CO, followed by LPS stimulation, on the hepatic mRNA expression of pro-inflammatory cytokines and ER stress-related genes. Compared with the HFD group, the mRNA levels of IL-1β and CXCL1 were significantly lower in the HFD + PO and HFD + CO groups (Figure 8a,b). In addition, the BiP mRNA levels in the HFD + PO and HFD + CO groups were also lower than those of the HFD group (Figure 8c); however, CHOP mRNA expression did not significantly differ among groups (Figure 8d).

### 3.9. Partial Replacement of Dietary Fat with PO and CO Suppressed the Nuclear Factor-Kappa B (NF-κB) and Mitogen-Activated Protein Kinase (MAPK) Signaling Pathways and Increased the Antioxidant Enzyme Expression in the Liver of LPS-Injected Rats

**Figure 9:**
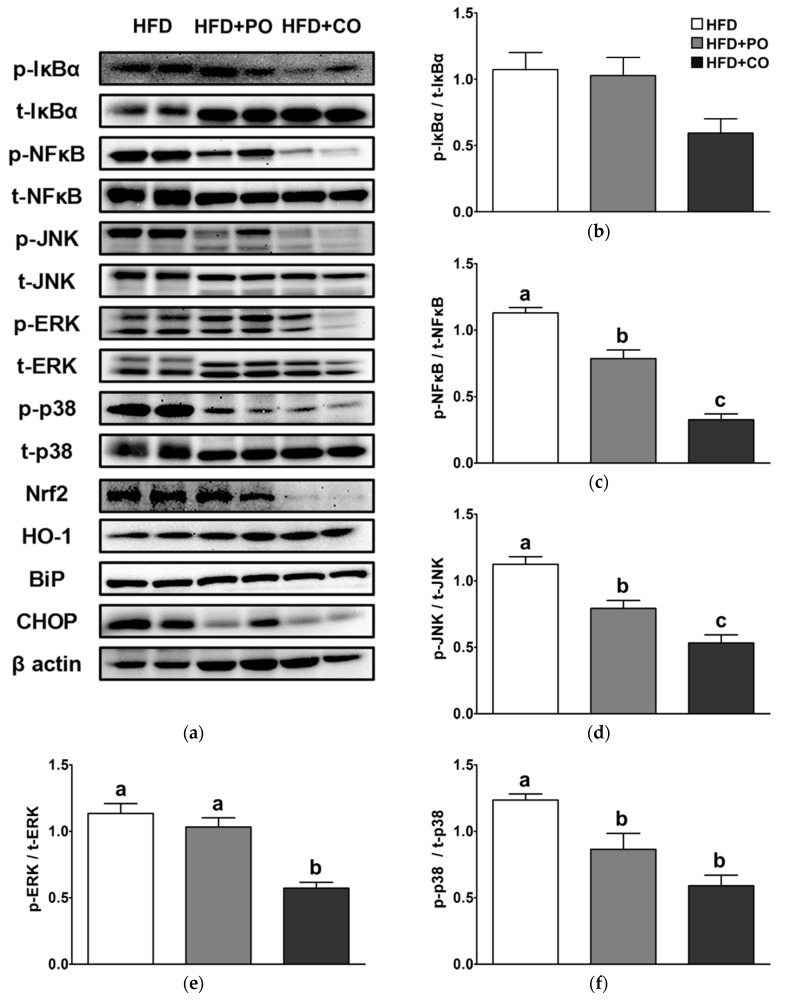

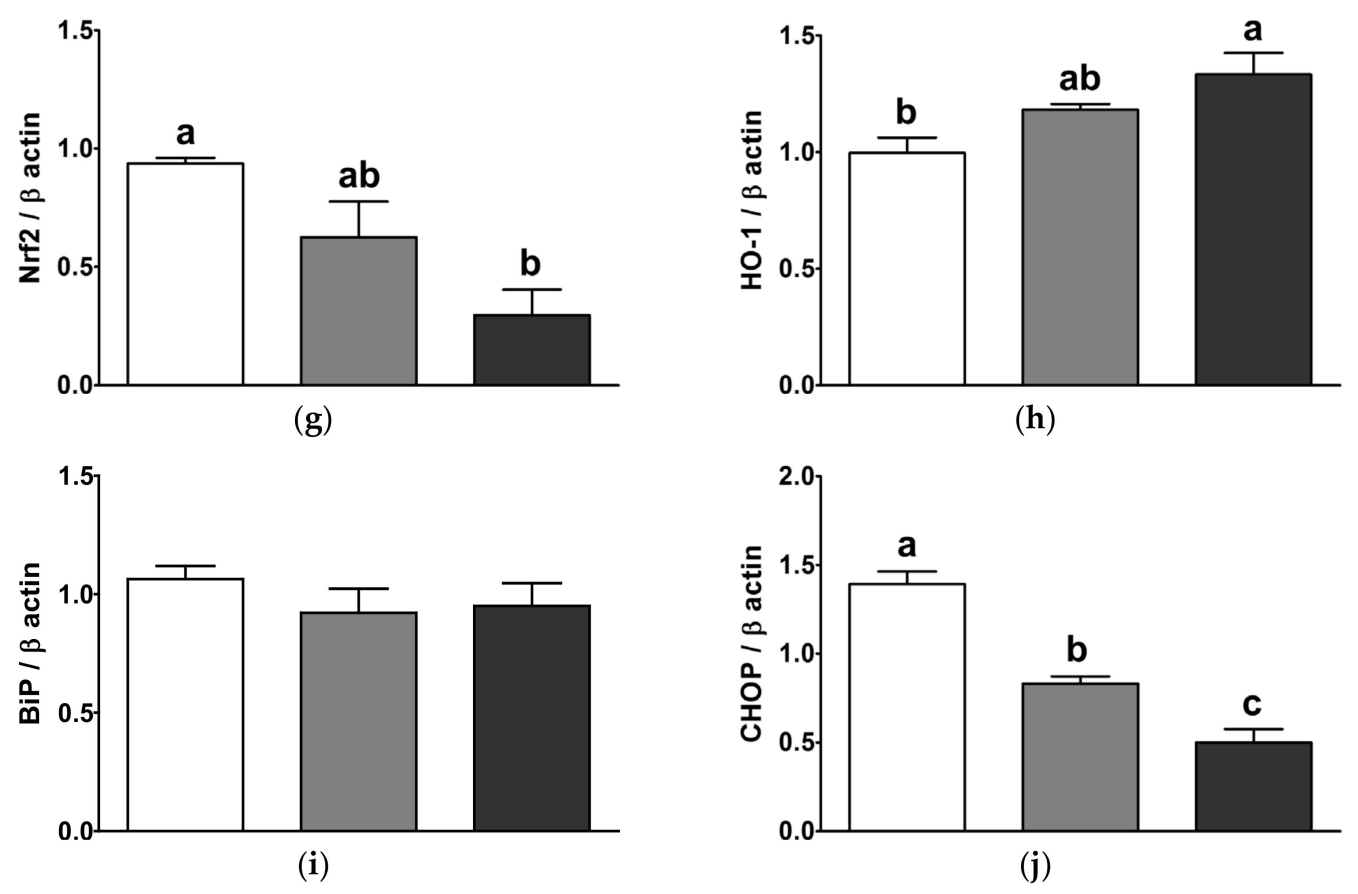
Effects of partial replacement of dietary fat with PO or CO and LPS stimulation on the levels of proteins related to the nuclear factor-kappa B (NF-kB) and mitogen-activated protein kinase (MAPK) pathways, oxidative stress, and ER stress in the liver. Five-week-old male Sprague-Dawley rats were fed either the HFD, HFD + PO, or HFD + CO for 12 weeks and then treated with LPS (5 mg/kg) for 24 h (*n* = 8 per group). (**a**) Representative Western blot images; (**b**) phospho-nuclear factor of kappa light polypeptide gene enhancer in B-cells inhibitor alpha (p-IkBα) level; (**c**) phospho-nuclear factor kappa-light-chain-enhancer of activated B cells (p-NF-κB) level; (**d**) phospho-c-Jun N-terminal kinases (p-JNK) level; (**e**) phospho-extracellular signal-regulated kinases (p-ERK) level; (**f**) phospho-p38 (p-p38) level; (**g**) nuclear factor erythroid 2-related factor 2 (Nrf2) level; (**h**) heme oxygenase 1 (HO-1); (**i**) binding immunoglobulin protein (BiP) level; (**j**) C/EBP homologous protein (CHOP) level. The expression of each protein was normalized to a value for β-actin, the internal control of protein content. Values are presented as the mean ± standard deviation. Data were analyzed using one-way ANOVA and Tukey’s multiple comparisons post hoc test for multiple comparisons. Means with different letters indicate significant differences at *p* < 0.05. LPS, lipopolysaccharide.

To elucidate the anti-inflammatory mechanism by which PO and CO exert and their relevance to oxidative stress and ER stress, we analyzed the expression of proteins in NF-κB/MAPK signaling pathways, antioxidation, and ER stress in the liver. The level of p-IκBα was not significantly altered by the dietary intervention (Figure 9a,b); however, the p-NF-κB level was significantly lower in the HFD + PO and HFD + CO groups than in the HFD group, and the level of the HFD + CO group was much lower than that of the HFD + PO group (Figure 9a,c). Similarly, the p-JNK level was significantly lower in the HFD + PO and HFD + CO groups than in the HFD group, and the level of the HFD + CO group was even lower than that of the HFD + PO group (Figure 9a,d). In the HFD + CO group, the p-ERK level was also significantly lower than in the HFD group, but not in the HFD + PO group (Figure 9a,e); however, the p-p38 MAPK level was significantly lower in the HFD + PO and HFD + CO groups than in the HFD group (Figure 9a,f). Compared with the HFD group, the Nrf2 level in the HFD + CO group was significantly lower, but the HO-1 level was significantly higher (Figure 9a,g,h). The HFD + PO group tended to have a lower Nrf2 level and higher HO-1 level than the HFD group, but the differences were not statistically significant (Figure 9a,g,h). The BiP level was not significantly changed by the diets (Figure 9a,i); however, the CHOP level was significantly lower in the HFD + PO and HFD + CO groups than in the HFD group, and the level of the HFD + CO group was much lower than that of the HFD + PO group (Figure 9a,j).

## 4. Discussion

Multiple researchers have demonstrated that SFAs initiate local inflammation and perturb metabolic homeostasis [23,42]. In contrast, PUFA supplementation has been reported to alleviate diet-induced inflammation, dyslipidemia, and insulin resistance in rodents [24,25]. Recently, it has been reported that acute inflammatory stimuli during high-fat diet consumption further exacerbate pro-inflammatory cytokine production and morphological changes in the liver [8,43]. Therefore, we aimed to investigate whether partial replacement of dietary fat with PUFAs alleviates LPS-induced inflammation in high-fat diet-induced obese rats. LPS is a structural component of the outer membrane of Gram-negative bacteria and is a very effective inflammatory inducer [44]. In a study by Imajo et al., mice fed an HFD for 12 weeks in combination with a single injection of LPS (0.25 mg/kg/day) showed the NASH-like aspect [6]; however, the effects of PUFA in rats injected with LPS during HFD consumption has not yet been well understood.

To replace SFAs with PUFAs, we supplemented a high-fat diet with PO or CO, rich sources of *n*-3 fatty acids and *n*-6 fatty acids, respectively. The doses of PO and CO used in this study were determined based on clinical requirements. The recommendations for the ratio of the daily intake of *n*-3 and *n*-6 fatty acids from diets established by different health agencies are 1:2–1:10 [31]. In previous studies, PO contained 60–70% of their fatty acids as *n*-3 fatty acids, while CO contained 50–60% of their fatty acids as *n*-6 fatty acids [45,46]. In this study, the percentage of fat in each diet was 23.9%, and the rate of *n*-3 fatty acids from total fat in the HFD + PO diet was 8.24%, while the *n*-6 fatty acid percentage from total fat in the HFD + CO diet was 28.4%. Therefore, we assumed that rats in the HFD + PO group consumed 0.10 g/day/100 g body weight of *n*-3 fatty acids while the rats in the HFD + CO group consumed 0.34 g/day/100 g body weight of *n*-6 fatty acids, as food intake of rats usually is 4–5 g/day/100 g body weight [47]. The Institute of Medicine (IOM) of the US recommends that men consume ~1.6 g/day of *n*-3 fatty acids and ~17 g/day of *n*-6 fatty acids, respectively [48]. When the human doses are translated into rat doses based on the body surface area, the doses in rats are ~0.02 g/day/100 g body weight of *n*-3 fatty acids and ~0.17 g/day/100 g body weight of *n*-6 fatty acids [32]. Therefore, the content of *n*-3 and *n*-6 fatty acids added to each experimental diet seemed to be slightly higher than the amount recommended to potential clinical implications. Consequently, the blood *n*-3 fatty acid concentration of the HFD + PO group was 3.7-fold higher than that of the HFD + CO group. On the other hand, the blood *n*-6 fatty acid concentration of the HFD + CO group was 1.4-fold higher than that of the HFD + PO group. These results indicate that the blood fatty acid profile is precisely reflected by the dietary fatty acid composition.

High energy intake from a high-fat diet is a significant cause of obesity [49]. Studies have also reported that body weight gain and body fat composition are affected by the fatty acid composition of dietary fat [50,51]. However, in the present study, there were no noticeable changes in body weight gain among the groups during the feeding period. Similarly, daily food intake was not affected by the diets, and thus, the food efficiency ratio did not differ among the experimental groups. These confirmatory results are supported by a study by Tian et al., suggesting that a high-fat diet combined with fish oil and perilla oil did not cause body weight changes [29]. Therefore, it is assumed that during HFD consumption, PUFA-rich diets may not be sufficient to alleviate weight increase.

Chronic excess energy intake has been shown to affect peripheral insulin resistance, leading to hyperinsulinemia [52]. However, *n*-3 PUFA has been reported to protect against insulin resistance in sucrose-fed rats [53]. On the other hand, it has been reported that high doses of PO (45% calories from PO) lead to insulin resistance, thus raising the potential risk of dysregulated glucose metabolism [54]. In our experimental settings, HFD + PO and HFD + CO diets did not alter the ability of rats to clear excess blood glucose, as shown in the OGTT results. Nevertheless, when rats were injected with LPS, serum insulin levels were increased; however, PO and CO decreased fasting blood glucose and insulin levels. According to previously reported data, the concentrations of serum glucose in male SD rats over 17 weeks of age were 141 ± 19 (ranges from 106 to 184) [55]. Therefore, it is implied that serum concentrations glucose was higher than normal levels in rats fed an HFD, whereas the LPS-treated rats supplemented with PO and CO recovered serum glucose concentrations to normal levels. Therefore, PO and CO eventually alleviated the increase in HOMA-IR induced by LPS. HOMA-IR is an indicator for assessing beta-cell function and insulin resistance in a steady state. A high HOMA-IR indicates that the cells require more insulin than normal to maintain their blood sugar balance. In a previous study, a high-fat diet was reported to elevate enterobacterial production and facilitate translocation of LPS into the systemic circulation [44]. Dietary fatty acids and LPS have been shown to promote insulin resistance through the activation of TLR4 [56]. The role of leptin in lowering glucose levels in animal models of insulin deficiency has been reported [57]. In contrast, in obesity, partial reduction of plasma leptin levels has been suggested to enhance insulin sensitivity [58]. In this study, we showed that experimental diets did not affect serum leptin levels, whereas stimulation with LPS significantly lowered serum leptin levels, demonstrating a close relationship between leptin and LPS-induced insulin resistance. These results were consistent with those reported by Al-Lahham et al., which indicated that LPS treatment suppressed leptin release from subcutaneous adipose tissue culture from obese patients [59]. Therefore, it is hypothesized that partial replacement of dietary fat with PO and CO may ameliorate dysregulated glucose metabolism induced by LPS stimulation in obesity. 

Chronic overnutrition has been viewed as a significant cause of obesity and dyslipidemia [60]. Dyslipidemia in obesity is characterized by high TG, TC, LDL-C, and low HDL-C levels, which significantly influence CVD risk [60]. LPS has also been reported to influence blood lipid profiles. In a previous study, LPS exposure during pregnancy resulted in dyslipidemia in offspring rats, and dysregulation of blood lipids in offspring exposed to LPS was more prominent following a high-fat diet [61]. Likewise, in this study, LPS stimulation promoted dyslipidemia due to a high-fat diet by increasing TC levels and decreasing non-HDL-C and HDL levels; therefore, the AC and CRF were remarkably higher in the LPS-stimulated rats. Meanwhile, in an epidemiological study, consumption of *n*-3 fatty acids was shown to have a strong inverse correlation with TG and a strong positive correlation with HDL-C [62]. However, in this study, to our surprise, PO and CO worsened LPS-induced dyslipidemia unexpectedly by increasing TG, TC, and non-HDL-C levels. Interestingly, the AST, ALT, and ALP levels associated with hepatic function showed a similar pattern to the indices related to dyslipidemia. In LPS-injected rats, AST, ALT, and ALP levels were increased, and PO and CO supplementation significantly increased these levels. In previous studies, dietary supplementation with *n*-3 PUFAs revealed no adverse effects following short-term administration [63,64]. However, Jenkinson et al. suggested that long-term supplementation with *n*-3 fatty acids may have adverse effects due to increased oxidative stress [65]. In contrast, the combined use of antioxidant fat-soluble vitamin E with *n*-3 fatty acids significantly lowered the AST, ALT, TG, and LDL-C levels [66]. Furthermore, vitamin E supplementation has been suggested to prevent PO oxidation, thus having a beneficial impact on hepatic function parameters in rats and reducing the risks of hepatic injury, glucose tolerance, and insulin resistance [27,65,66]. The content of vitamin E in our experimental diets was 75 IU/kg diet according to the recommendation of the Amirian Institute of Nutrition (AIN) committee [33]. However, dietary vitamin E requirements increase with increased consumption of dietary PUFAs due to susceptibility to fatty acid oxidation. Indeed, in a clinical trial, people who had taken additional *n*-3 fatty acids showed lower plasma α-tocopherol levels compared to their placebo group [67]. Moreover, in dogs fed a diet enriched with fish oil for 8 weeks, the level of plasma α-tocopherol was lowered [68]. In particular, levels of α-tocopherol decreased in the plasma and liver of rats fed a diet rich in *n*-3 fatty acids, while in the heart where *n*-3 fatty acids are deposited, α-tocopherol concentration increased [69]. Therefore, we postulated that some negative results in rats fed a diet supplemented with PO and CO in our study are presumably due to the increased demand for antioxidant activity; thus, we carefully suggest that it would be better to take sufficient antioxidants in a diet high in PUFA.

A previous study has shown that when people are overfed with HFD, the ratio of lean tissue to adipose tissue is higher in people with a PUFA-enriched diet than in those who consume high SFA, although the weight gain in both groups was almost the same [70]. However, in the present study, both HFD + PO and HFD + CO diets did not affect WAT fat pad weights, implying that partial replacement of dietary fat with PO and CO was insufficient to alter adiposity due to HFD. On the other hand, the liver weight tended to be increased by LPS, whereas CO supplementation reduced the weight of the liver regardless of LPS stimulation. The weight increase in the liver is caused by an accumulation of lipids or a result of cell damage, hepatocellular hypertrophy, or hyperplasia [71]. Indeed, the hepatic levels of TG were higher in rats injected with LPS; however, PO and CO lowered the level of hepatic TG, especially when rats were injected with LPS. Consistent with our results, LPS accelerated the progression of hepatic steatosis in rats [72]. In contrast, *n*-6 PUFAs have been reported to reduce liver fat in overweight individuals compared to SFA [73]. Moreover, Kim et al. reported that PO significantly reduced hepatic TG levels induced by HFD [74]. An increasing body of evidence has suggested that hepatic fat accumulation is associated with insulin resistance, suggesting that the health benefits of PO and CO might be associated with lower hepatic fat accumulation. Interestingly, the TG level of EAT showed an opposite pattern to that of the liver. LPS induced TG reduction in the EAT, while PO and CO increased the TG decrease caused by LPS. Studies have reported that, in obese conditions, enlarged adipocytes lose their ability to store energy, resulting in the release of fatty acids, which are absorbed into the liver [75]. Thus, we assumed that the weight decrease of EAT TG might be related to the increased lipolysis from excessive fat storage, and the increase in hepatic TG accumulation might be related to the increased circulating free fatty acids and uptake to the liver. 

Nonalcoholic steatohepatitis is a type of NAFLD that occurs when excess fat accumulation in the liver is accompanied by liver inflammation [6]. It has been well proven in numerous cell types and animal models that PUFAs have a beneficial effect on health by diminishing the secretion of pro-inflammatory cytokines [76,77,78,79]. On the other hand, some studies have reported that SFAs promote inflammation by increasing the secretion of pro-inflammatory cytokine [80,81,82]. Therefore, we further analyzed the mRNA levels of inflammatory molecules and signaling pathways involved in inflammation to observe whether PUFAs improve hepatic inflammation in an HFD and LPS-induced steatohepatitis rat model. In this study, PO and CO significantly downregulated the mRNA expression levels of IL-1β and CXCL1, indicating that PO and CO efficiently inhibited inflammatory responses in the liver. Our findings were consistent with those of Xu et al., who observed that PO extract lowered the expression of pro-inflammatory cytokines [83]. Previous studies have reported that LPS/TLR4 downstream signaling pathways lead to gene transcription of pro-inflammatory cytokines and chemokines, including interleukin-1β (IL-1β) and chemokine (C-X-C motif) ligand 1 (CXCL-1) through the activation of NF-κB and MAPK signaling pathways [84,85,86]. In resting states, NF-κB is sequestered in the cytosol by nuclear factor of kappa light polypeptide gene enhancer in B-cells inhibitor, alpha (IκBα). Phosphorylation of the IKK complexes by TAK1 results in their proteasomal degradation of IκBα, and liberation of NF-κB subsequently translocates from the cytosol to the nucleus to induce gene expression [86]. Concurrently, TAK1 activates the MAPK family, including Jun amino-terminal kinases (JNK), extracellular signal-regulated kinases (ERKs), and p38. The phosphorylation of MAPK proteins results in activated activator protein 1 (AP-1), which translocates to the nucleus [87]. Therefore, we concluded from these results that PO and CO could exert effects on inhibit inflammation in rats injected with LPS during HFD feeding, which may be partially associated with the inactivation of NF-κB/MAPK signaling pathways.

Increased uptake of fatty acids in the liver could induce hepatic lipotoxicity by activating ER stress signaling pathways, known as the UPR [5,6,7,8]. Persistent activation of the UPR was shown to generate reactive oxygen species, eliciting inflammatory responses [6]; therefore, reducing ER stress and concurrent oxidative stress could be a potential therapeutic mechanism for preventing inflammation during HFD consumption. Many studies have suggested that BiP and CHOP proteins, markers of elevated ER stress, are increased in the liver due to obesity [88,89]. BiP is a molecular chaperone that plays a role in enhancing cellular folding capacity, but its synthesis is markedly induced under conditions that lead to the accumulation of unfolded polypeptides in the ER. CHOP is a member of the C/EBP family of nuclear proteins caused by ER stress, which mediates apoptosis. In the present study, PO and CO diets downregulated the mRNA expression of BiP and CHOP protein levels in the liver, indicating that PO and CO supplementation effectively ameliorated ER stress response. These results were consistent with a study by Bae et al., who reported that PO consumption attenuates ER stress markers [90]. In the present study, we also analyzed the protein expression of Nrf2 and HO-1 to investigate whether PO and CO could improve antioxidant potential. Nrf2 is a crucial transcription factor found primarily in the liver. In response to oxidative stress, Nrf2 is translocated to the nucleus to induce transcription of various antioxidant and detoxification-related genes [91]. HO-1 is an enzyme that catalyzes heme degradation to produce biliverdin, which possesses antioxidant properties. In our study, the livers of PO-and CO-supplemented rats had lower Nrf2 and higher HO-1 levels. We assumed that increased HO-1 implies greater antioxidant capacity, and the lowered Nrf2 might be possibly due to the reduced oxidative stress stimuli. Therefore, these results indicate that hepatic ER stress and antioxidant capacity were increased by the HFD + PO and HFD + CO diets in rats injected with LPS, which may be associated with the anti-inflammatory effects of PO and CO.

## 5. Conclusions

In conclusion, partial replacement of PO and CO improved insulin resistance, hepatic steatosis, and hepatic inflammation induced by LPS in rats fed an HFD. Furthermore, the anti-inflammatory effects of PO and CO were partially mediated by the inhibition of NF-κB/MAPK signaling pathways, ER stress, and an increase in antioxidant capacity. Therefore, it is assumed that supplementation with PO and CO could be an effective strategy to alleviate the metabolic dysregulation caused by hepatic inflammation during HFD consumption. The results of this study can be used as the basis for future clinical trials, and it is expected that the effects of PO and CO in regulating physiological activity could become clearer through additional clinical studies.

## Figures and Tables

**Table 1:** Constant ingredients in the three experimental diets.

Ingredient (g/kg)	Amount
Casein	220
L-cysteine	3.4
Sucrose	100
Corn starch	160
Dextrose	155
Cellulose	58
Mineral mix ^1^	43
Vitamin mix ^2^	19
Choline bitartrate	2.8
Lard	55
*tert*-Butylhydroquinone	0.034
Energy (kcal/g)	4.8
Fat (%)	23.9
Fat (kcal%)	45

^1^ AIN-93G Mineral mix [33]. ^2^ AIN-93 Vitamin mix [33].

**Table 2:** Variable ingredients in the three experimental diets.

Ingredient (g/kg)	HFD	HFD + PO	HFD + CO
Butter ^1^	184	164	104
Perilla oil	0	20	0
Corn oil	0	0	80

^1^ Unsalted pure butter (Anchor brand, Fonterra Ltd., Hamilton, New Zealand). Abbreviations: HFD, high-fat diet; HFD + PO, high-fat diet + perilla oil; HFD + CO, high-fat diet + corn oil.

**Table 3:** Quantitative reverse transcription-polymerase chain reaction (qRT-PCR) primer sequences.

Gene	Primer Sequence (5′→3′)	
*CXCL-1*	Forward Reverse	CCACACTCAAGAATGGTCGC GTTGTCAGAAGCCAGCGTTC
*IL-1β*	Forward Reverse	AAAAATGCCTCGTGCTGTCT TCGTTGCTTGTCTCTCCTTG
*BiP*	Forward Reverse	TGCCCACCAAGAAGTCTCAGA TCAAATGTACCCAGAAGGTGATTG
*CHOP*	Forward Reverse	GGAGAAGGAGCAGGAGAATG GAGACAGACAGGAGGTGATG
*GAPDH*	Forward Reverse	CTGTGTCTTTCCGCTGTTTTC TGTGCTGTGCTTATGGTCTCA

Abbreviations: CXCL-1, chemokine (C-X-C motif) ligand 1; IL-1β, interleukin 1β; BiP, binding immunoglobulin protein; CHOP, C/EBP homologous protein; GAPDH, glyceraldehyde 3-phosphate dehydrogenase.

**Table 4:** List of antibodies for western blot analysis.

Antibody	Company	Catalog Number	Dilution
p-IκBα	Cell Signaling	2859	1:1000
t-IκBα	Cell Signaling	4814	1:1000
p-NFκB	Cell Signaling	3033	1:1000
t-NFκB	Cell Signaling	8242	1:1000
p-JNK	Cell Signaling	9251	1:500
t-JNK	Cell Signaling	9252	1:1000
p-ERK	Cell Signaling	4370	1:3000
t-ERK	Cell Signaling	4695	1:1000
p-p38	Cell Signaling	4511	1:1000
t-p38	Cell Signaling	8690	1:1000
Nrf2	Cell Signaling	12721S	1:1000
HO-1	Cell Signaling	5853S	1:1000
BiP	Cell Signaling	3183	1:1000
CHOP	Cell Signaling	2895	1:1000
β-actin	Santa Cruz	sc-47778	1:2000
Anti-rabbit IgG	Cell Signaling	7074	1:3000
Anti-mouse IgG	Cell Signaling	7076	1:1000

Abbreviations: IkBα, nuclear factor of kappa light polypeptide gene enhancer in B-cells inhibitor alpha; NF-κB, nuclear factor kappa-light-chain-enhancer of activated B cells; JNK, c-Jun N-terminal kinases; ERK, extracellular signal-regulated kinases; Nrf2, nuclear factor erythroid 2-related factor 2; HO-1, heme oxygenase 1; BiP, binding immunoglobulin protein; CHOP, C/EBP homologous protein.

**Table 5:** Summary of statistical analysis by two-way analysis of variance (ANOVA) for main effects and interactions.

Parameter	LPS Main Effect	Diet Main Effect	LPS X Diet Interaction
Serum glucose, insulin, and leptin levels			
Glucose	**** *p* < 0.0001	**** *p* < 0.0001	* *p* < 0.05
Insulin	**** *p* < 0.0001	** *p* < 0.01	** *p* < 0.01
HOMA-IR	**** *p* < 0.0001	**** *p* < 0.0001	**** *p* < 0.0001
Serum leptin	**** *p* < 0.0001	ns *p* = 0.328	ns *p* = 0.332
Serum lipid profile			
Triglyceride	**** *p* < 0.0001	*** *p* < 0.001	**** *p* < 0.0001
Total cholesterol	**** *p* < 0.0001	**** *p* < 0.0001	*** *p* < 0.001
HDL-cholesterol	**** *p* < 0.0001	ns *p* = 0.191	ns *p =* 0.573
Non-HDL-cholesterol	**** *p* < 0.0001	**** *p* < 0.0001	**** *p* < 0.0001
Atherogenic coefficient	**** *p* < 0.0001	** *p* < 0.01	* *p* < 0.05
Cardiac risk factor	**** *p* < 0.0001	** *p* < 0.01	* *p* < 0.05
Hepatic function parameters			
Serum AST	**** *p* < 0.0001	**** *p* < 0.0001	**** *p* < 0.0001
Serum ALT	**** *p* < 0.0001	**** *p* < 0.0001	**** *p* < 0.0001
Serum ALP	**** *p* < 0.0001	**** *p* < 0.0001	**** *p* < 0.0001
Relative tissue weights			
Liver	ns *p* = 0.972	** *p* < 0.01	ns *p* = 0.134
WAT	ns *p =* 0.610	ns *p* = 0.384	ns *p* = 0.846
EAT	ns *p* = 0.718	ns *p* = 0.420	ns *p* = 0.412
MAT	ns *p* = 0.102	ns *p* = 0.074	** *p* < 0.01
RAT	ns *p* = 0.484	ns *p* = 0.861	ns *p* = 0.523
PAT	ns *p* = 0.319	ns *p* = 0.465	ns *p* = 0.541
Lipid contents in liver and epididymal adipose tissue			
Hepatic triglyceride	**** *p* < 0.0001	**** *p* < 0.0001	**** *p* < 0.0001
Hepatic total cholesterol	**** *p* < 0.0001	*** *p* < 0.001	ns *p* = 0.846
EAT triglyceride	ns *p* = 0.160	**** *p* < 0.0001	**** *p* < 0.0001
EAT total cholesterol	* *p* < 0.05	**** *p* < 0.0001	**** *p* < 0.0001

* *p* < 0.05, ** *p* < 0.01, *** *p* < 0.001, **** *p* < 0.0001; ns, not significant. Abbreviations: WAT, white adipose tissue; EAT, epididymal adipose tissue; MAT, mesenteric adipose tissue; RAT, retroperitoneal adipose tissue; PAT, perirenal adipose tissue; HOMA-IR, homeostasis model assessment of insulin resistance; HDL, high-density lipoprotein; AST, aspartate aminotransferase; ALT, alanine aminotransferase; ALP, alkaline phosphatase; CXCL-1, chemokine (C-X-C motif) ligand 1; IL-1β, interleukin 1β; BiP, binding immunoglobulin protein; CHOP, C/EBP homologous protein.

**Table 6:** Fatty acid composition of experimental diets.

Fatty Acid (%)	HFD	HFD + PO	HFD + CO
Palmitic acid (C16:0)	59.64	35.27	25.46
Stearic acid (C18:0)	10.32	12.44	9.36
Oleic acid (C18:1*n*-9c)	23.77	33.21	33.53
Elaidic acid (C18:1*n*-9t)	1.45	2.8	1.44
Linoleic acid (C18:2*n*-6c)	4.82	7.58	28.08
Linolelaidic acid (C18:2*n*-6t)	<LLOQ	0.27	0.33
α-linolenic acid (C18:3*n*-3)	<LLOQ	8.12	0.38
Eicosanoic acid (C20:0)	<LLOQ	0.2	<LLOQ
Eicosenoic acid (C20:1*n*-9)	<LLOQ	<LLOQ	1.36
Eicosatrienoic acid (C20:3*n*-3)	<LLOQ	0.12	0.08
SFA	69.96	47.91	34.81
MUFA	25.23	36.01	36.33
*trans*	1.45	3.06	1.77
PUFA	4.82	16.09	28.86
*n*-6	4.82	7.85	28.40
*n*-3	<LLOQ	8.24	0.46
*n*-6/*n*-3	NC	0.95	62.24

Abbreviations: HFD + PO, high-fat diet + perilla oil; HFD + CO, high-fat diet + corn oil; SFA, saturated fatty acids; MUFA, monounsaturated fatty acids; TFA, trans-fatty acids; PUFA, polyunsaturated fatty acids; <LLOQ, lower than the lower limit of quantification; NC, not calculated.

**Table 7:** Fatty acid composition of the whole blood of rats.

Fatty Acid (%)	HFD	HFD + PO	HFD + CO
Myristic acid (C14:0)	0.64 ± 0.32 ^ns^	0.70 ± 0.10	0.58 ± 0.11
Palmitic acid (C16:0)	26.40 ± 1.70 ^ns^	26.60 ± 0.20	25.73 ± 1.17
Palmitoleic acid (C16:1*n*-7c)	1.34 ± 0.29 ^a^	0.82 ± 0.22 ^ab^	0.42 ± 0.16 ^b^
Palmitoleic acid (C16:1*n*-7t)	0.39 ± 0.12 ^a^	0.19 ± 0.02 ^b^	0.17 ± 0.05 ^b^
Stearic acid (C18:0)	14.65 ± 0.35 ^b^	18.57 ± 0.31 ^a^	18.17 ± 1.67 ^a^
Oleic acid (C18:1*n*-9c)	19.50 ± 1.90 ^a^	12.93 ± 1.33 ^b^	10.28 ± 0.69 ^b^
Oleic acid (C18:1*n*-9t)	0.38 ± 0.26 ^ns^	0.47 ± 0.12	0.40 ± 0.22
Linoleic acid (C18:2*n*-6c)	11.47 ± 1.53 ^a^	7.23 ± 0.39 ^b^	9.91 ± 0.85 ^a^
Linoleic acid (C18:2*n*-6t)	0.07 ± 0.01 ^b^	0.06 ± 0.01 ^b^	0.10 ± 0.02 ^a^
α-linolenic acid (C18:3*n*-3)	0.25 ± 0.02 ^b^	0.37 ± 0.03 ^a^	0.04 ± 0.02 ^c^
γ-linolenic acid (C18:3*n*-6)	0.10 ± 0.07 ^ns^	0.03 ± 0.02	0.04 ± 0.02
Eicosenoic acid (C20:1*n*-9)	0.23 ± 0.06 ^ns^	0.11 ± 0.02	0.13 ± 0.08
Eicosadienoic acid (C20:2*n*-6)	0.31 ± 0.10 ^ns^	0.26 ± 0.10	0.35 ± 0.07
Dihomo-y-linolenic acid (C20:3*n*-6)	1.15 ± 0.57 ^ns^	0.91 ± 0.12	0.40± 0.10
Arachidonic acid (C20:4*n*-6)	16.70 ± 1.90 ^c^	20.63 ± 1.45 ^b^	26.33 ± 0.85 ^a^
Eicosapentaenoic acid (C20:5*n*-3)	0.34 ± 0.07 ^b^	1.89 ± 0.23 ^a^	0.23 ± 0.07 ^b^
Docosatetraenoic acid (C22:4*n*-6)	0.96 ± 0.09 ^b^	0.44 ± 0.06 ^c^	2.50 ± 0.22 ^a^
Docosapentaenoic acid (C22:5*n*-3)	0.84 ± 0.10 ^b^	2.98 ± 0.26 ^a^	0.65 ± 0.14 ^b^
Docosahexaenoic acid (C22:6*n*-3)	3.65 ± 0.88 ^a^	4.08 ± 0.83 ^a^	1.60 ± 0.26 ^b^
Docosapentaenoic acid (C22:6*n*-6)	0.20 ± 0.01 ^b^	0.11 ± 0.05 ^b^	1.34 ± 0.52 ^a^
Lignoceric acid (C24:0)	0.16 ± 0.11 ^b^	0.33 ± 0.01 ^a^	0.26 ± 0.04 ^ab^
Nervonic acid (C24:1n-9)	0.12 ± 0.08 ^ns^	0.14 ± 0.02	0.15 ± 0.05
SFA	41.84 ± 1.77 ^b^	46.19 ± 0.18 ^a^	44.74 ± 0.49 ^a^
MUFA	21.95 ± 2.35 ^a^	14.66 ± 1.55 ^b^	11.55 ± 0.73 ^b^
TFA	0.83 ± 0.14 ^ns^	0.72 ± 0.12	0.67 ± 0.16
PUFA	36.01 ± 4.09 ^b^	38.99 ± 1.58 ^ab^	43.49 ± 0.35 ^a^
*n*-6	30.95 ± 3.26 ^b^	29.67 ± 1.34 ^b^	40.96 ± 0.28 ^a^
*n*-3	5.07 ± 0.83 ^b^	9.32 ± 1.26 ^a^	2.52 ± 0.11 ^c^
*n*-6/*n*-3	6.15 ± 0.37 ^b^	3.22 ± 0.47 ^c^	16.25 ± 0.66 ^a^

Values are presented as the mean ± standard deviation (*n* = 3 per group). Fatty acid composition of whole blood was analyzed using pooled samples generated from 2–3 samples in each group due to the sample volume limitations. Data were analyzed using one-way analysis of variance followed by Tukey’s post hoc comparison. Means labeled without a common letter differ significantly (*p* < 0.05); ns, not significant. Abbreviations: HFD + PO, high-fat diet + perilla oil; HFD + CO, high-fat diet + corn oil; SFA, saturated fatty acids; MUFA, monounsaturated fatty acids; TFA, trans-fatty acids; PUFA, polyunsaturated fatty acids.

## Data Availability

The data presented in this study are available from the corresponding author upon request.
